# Recurrent pyoderma gangrenosum precipitated by breast cancer: a case report and review of the literature

**DOI:** 10.1186/1752-1947-8-226

**Published:** 2014-06-25

**Authors:** Renata Duchnowska, Ewa Ziajka, Agnieszka Góralska, Bartłomiej Grala

**Affiliations:** 1 Department of Oncology, Military Institute of Medicine, Szaserów 128 Street, 04-141 Warsaw, Poland; 2 Department of Dermatology, Military Institute of Medicine, Szaserów 128 Street, 04-141 Warsaw, Poland; 3 Department of Pathology, Military Institute of Medicine, Szaserów 128 Street, 04-141 Warsaw, Poland

**Keywords:** Pyoderma gangrenosum, Breast cancer, Skin ulceration

## Abstract

**Introduction:**

Pyoderma gangrenosum is a rare clinical entity of poorly understood pathogenesis, characterized by rapidly progressing skin necrosis. In around half of patients pyoderma gangrenosum is a manifestation of underlying systemic diseases, such as rheumatoid arthritis, inflammatory bowel disease or myeloproliferative disorders. There have been very few reports on the association of pyoderma gangrenosum with solid malignancies.

**Case presentation:**

We report a case of a 68-year-old Caucasian woman in whom pyoderma gangrenosum first appeared around 30 years earlier, at the time of exacerbation of rheumatoid arthritis, and recurred as a manifestation of locally advanced breast cancer. The causative role of the neoplastic process was partly confirmed by the healing of the skin ulceration only following effective endocrine cancer therapy, whereas earlier attempts with standard anti-inflammatory therapy were unsuccessful.

**Conclusions:**

Pyoderma gangrenosum has a recurrent nature and may be reactivated by various causes within several years. Therefore, a prompt and thorough diagnosis accompanied by treatment of the underlying disease is necessary.

## Introduction

Pyoderma gangrenosum is a rare clinical condition of poorly understood pathogenesis, characterized by rapidly progressing skin necrosis. In around half of patients, this entity is a manifestation of underlying systemic diseases, such as rheumatoid arthritis, inflammatory bowel disease or myeloproliferative disorders. There have been very few reports on the association of pyoderma gangrenosum with solid malignancies. We present a case of a 68-year-old woman in whom pyoderma gangrenosum first appeared around 30 years earlier, at the time of exacerbation of rheumatoid arthritis, and recurred as a manifestation of locally advanced breast cancer.

## Case presentation

**Figure 1:**
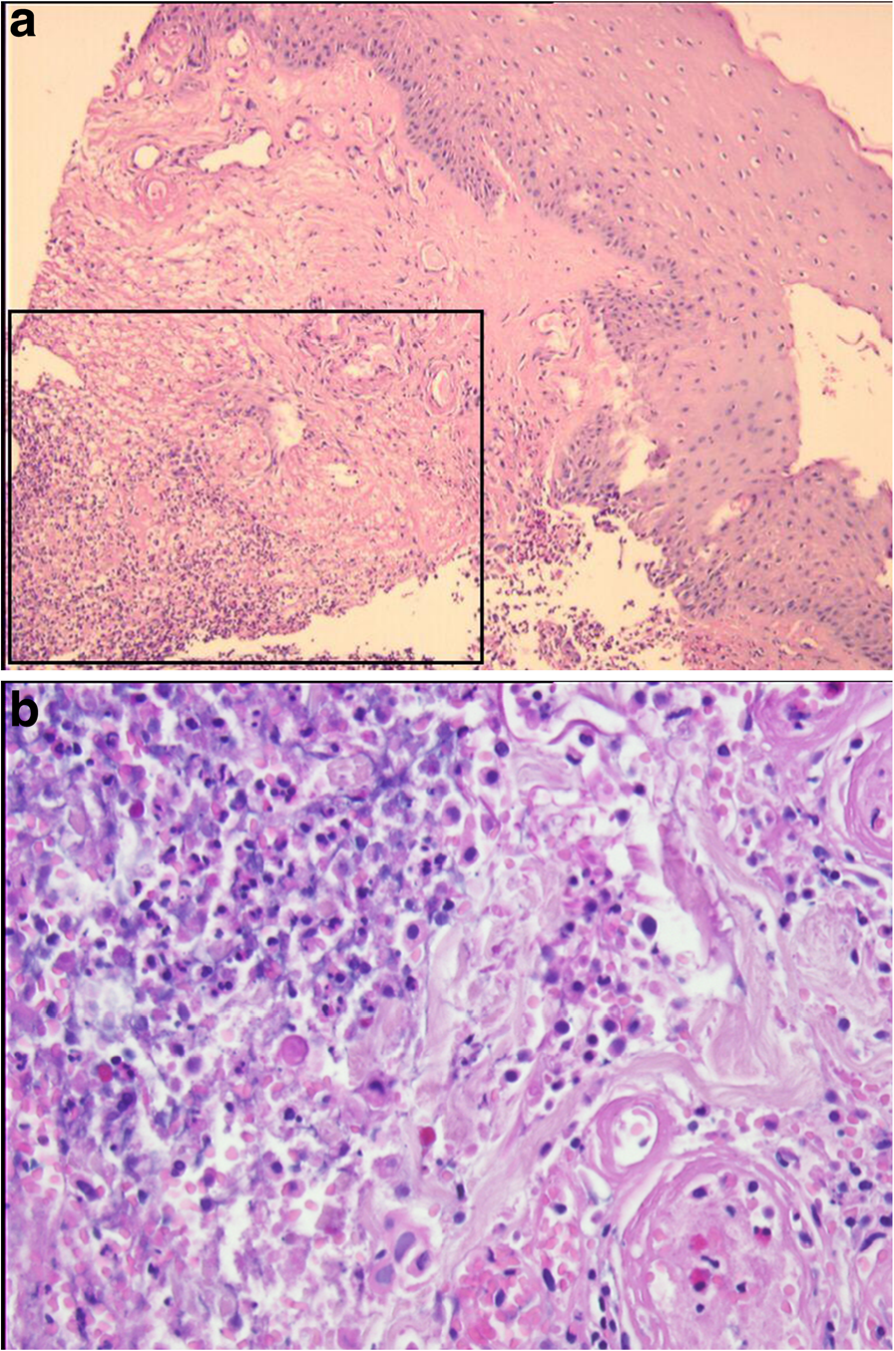
**Pyoderma gangrenosum. (a)** Biopsy specimen from the edge of the leg ulceration: histological pattern (hematoxylin and eosin stain (HE) staining; 200×). The inset within Figure 1a refers to Figure 1b. **(b)** An intensive mixed inflammatory infiltrate including mainly neutrophils, with accompanying vessel destruction and necrosis of the epithelium.

**Figure 2:**
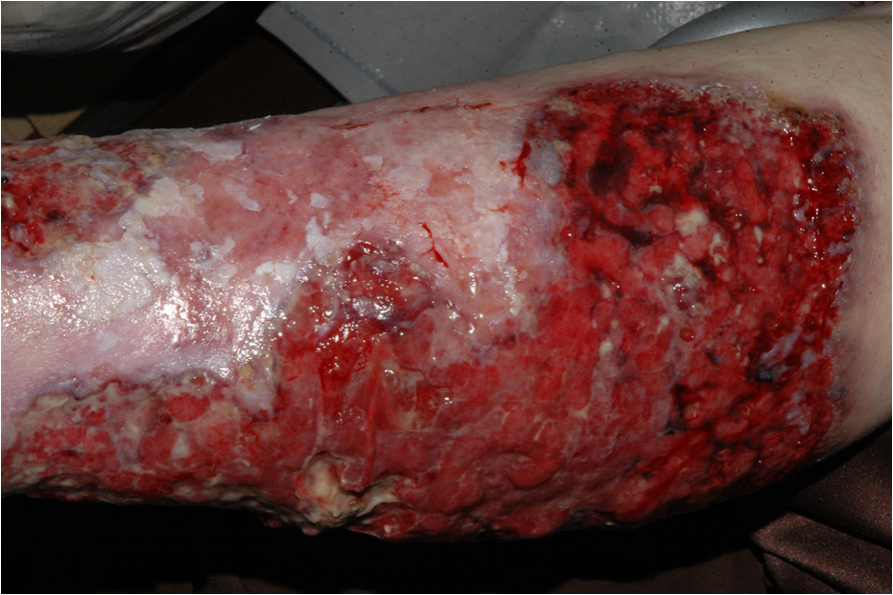
Ulceration of the left leg (at the time of initiation of induction endocrine therapy).

**Figure 3:**
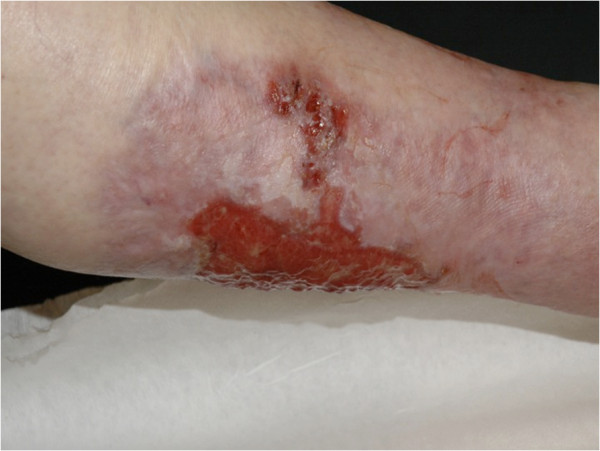
A continuous healing of the leg ulceration: after eight months of dermatology and oncology combination therapy.

A 68-year-old Caucasian woman presented with an ulceration of the anterior surface of her left leg. Thirty years earlier, at exacerbation of rheumatoid arthritis, our patient underwent therapy for a similar lesion located on her left forearm, diagnosed as pyoderma gangrenosum. Since then, the rheumatoid arthritis had been quiescent and had not required any therapy, and at admission there were no signs of recurrence. A physical examination showed an ulcerating tumor involving the lower quadrants of her left breast. The breast lump had appeared four years earlier, progressed rapidly within the last month and at diagnosis was clinically staged at T4aN1M0. A histopathology examination showed invasive ductal breast carcinoma, with estrogen receptor expression in 90 percent of the tumor cells, and no expression of progesterone receptor and epidermal growth factor receptor type 2 (HER2). A biopsy specimen from the edge of the leg ulceration showed pyoderma gangrenosum: an intensive mixed inflammatory infiltrate including mainly neutrophils, with accompanying vessel destruction and necrosis of the epithelium (Figure 1a,b). First-line therapy for pyoderma gangrenosum included a combination of dapsone (100mg/d orally) and prednisone (an increasing dose of 20 to 30mg/d orally), accompanied by a topical application of 2 percent detreomycin ointment and silicon dressing. After four months, due to further progression, cyclosporine administration was introduced at a starting dose of 300mg/d, decreasing to 100mg/d. Therapy was stopped after three months due to nephrotoxicity and microcytic anemia. Subsequent immunosuppressive therapy included sulfasalazin (3g/d orally) accompanied by topical management with potassium permanganate and 5 percent detreomycin ointment. Due to social factors, breast cancer treatment was initiated only five months after the diagnosis of pyoderma gangrenosum. Since our patient was diagnosed with strongly estrogen receptor-positive breast cancer with no life-threatening symptoms, a typical first-line induction endocrine therapy with aromatase inhibitor - letrozole - at a daily dose of 2.5mg was introduced. By this time, the leg ulceration had reached its largest dimension (Figure 2). A partial response of the breast tumor was accompanied by continuous healing of her leg ulceration (Figure 3) and after 12 months, the lesion completely resolved with scarring. As our patient refused mastectomy, a wide tumor excision was performed, however, postoperative radiotherapy was not applied due to a concern of excessive skin toxicity and activation of pyoderma gangrenosum in the irradiated area. After surgery, our patient finished five years of adjuvant letrozole therapy with complete response. Her leg ulceration has not recurred.

## Discussion

Pyoderma gangrenosum is a rare dermatosis characterized by rapidly progressive skin ulceration. The etiology of this entity is poorly understood but neutrophil and monocyte dysfunctions (defects in phagocytosis and chemotaxis) have been reported [1-6]. In around half of cases, pyoderma gangrenosum is associated with various underlying specific conditions, most frequently rheumatoid arthritis, inflammatory bowel disease or myeloproliferative disorders. There have been only a few reports on the association of pyoderma gangrenosum with solid malignancies including breast cancer [7-11]. Additionally, a few cases of pyoderma gangrenosum of the breast precipitated by breast surgery were reported [11-13]. The most common location of pyoderma gangrenosum is the legs but the disease may also involve other regions. Differential diagnosis includes Sweet’s syndrome, characterized by similar histologic features, fungal infections and inflammatory diseases of subcutaneous tissue [2,4,6]. The recommended first-line treatment is steroids or other immunosuppressive drugs, such as cyclosporine, azathiopirine, mycophenolate mofetil, cyclolophosfamide, chlorambucil or thalidomide [14]. Surgery is contraindicated due to pathergy, which is characterized by the development of new lesions at the site of even minor trauma [13]. Importantly, pyoderma gangrenosum management should be accompanied by treatment of the underlying disease, although surgery or radiotherapy may induce rapid development of ulceration at the site of therapeutic intervention [7,11-13].

In our patient, pyoderma gangrenosum was most likely induced by progressing breast cancer. The causative role of the neoplastic process was partly confirmed by the healing of the skin ulceration only following effective endocrine cancer therapy, whereas earlier attempts with corticosteroids were unsuccessful. The lack of improvement with corticosteroids has been atypical, as this therapy usually induces dramatic response within the first two weeks [14]. Another interesting feature of this case was the recurrent nature of pyoderma gangrenosum, with its first appearance 30 years earlier, during an exacerbation of rheumatoid arthritis. Thus, probably this entity may be reactivated by various causes within several years.

## Conclusions

Due to association with serious underlying conditions, rapid development and potentially serious consequences, pyoderma gangrenosum necessitates prompt and thorough diagnosis, including the search for coexisting disease [15,16]. If the latter is recognized, therapy should address both pyoderma gangrenosum and its causative factor.

## Consent

Written informed consent was obtained from the patient for publication of this case report and any accompanying images. A copy of the written consent is available for review by the Editor-in-Chief of this journal.

## Abbreviations

HER2: epidermal growth factor receptor type 2.

## Competing interests

The authors declare that they have no competing interests.

## Authors’ contributions

RD treated the patient and drafted the manuscript. EZ treated the patient and drafted the manuscript. AG treated the patient and drafted the manuscript. BG carried out the immunoassays and drafted the manuscript. All authors read and approved the final manuscript.
